# Tremor as a Rare Manifestation of Cefepime Neurotoxicity

**DOI:** 10.7759/cureus.59518

**Published:** 2024-05-02

**Authors:** Brittany Guidos, Kurt Lee, Elsa Tchouambou, Nathan Zaher

**Affiliations:** 1 Neurology, HCA Florida Westside Hospital, Plantation, USA; 2 Internal Medicine, HCA Florida Westside Hospital, Plantation, USA

**Keywords:** case report, adverse drug reactions, tremor, neurotoxicity, cefepime

## Abstract

The antibiotic cefepime is a fourth-generation cephalosporin with extended-spectrum coverage against both gram-positive and negative bacteria. It is commonly used in the inpatient setting to treat community-acquired pneumonia or urinary tract infection and has side effects, including diarrhea, nausea, vomiting, pruritus, headache, and, more rarely, hypersensitivity reactions or neurotoxicity. The current report is about an 88-year-old female patient who was brought to the hospital by her daughter due to an acute change in mental status resulting from a urinary tract infection. The patient received intravenous cefepime and subsequently developed a low-frequency tremor after one day of treatment. Cefepime was discontinued with a resolution of tremor in three days. Though neurotoxicity has been documented as a serious adverse event with cefepime, tremor is not one of the known neurotoxic manifestations. This patient is the first reported to develop a tremor as a neurotoxic side effect from taking cefepime. Healthcare providers should be aware of this potential side effect and may consider discontinuing treatment with cefepime if their patient develops a new tremor within days of initiating treatment.

## Introduction

The antibiotic cefepime is a fourth-generation cephalosporin with extended-spectrum coverage against both gram-positive and negative bacteria. This beta-lactam antibiotic has activity against multiple drug-resistant organisms, such as Pseudomonas aeruginosa, as well as Staphylococcus aureus, Streptococcus pneumoniae, and Enterobacteriaceae [1]. It is commonly used in the inpatient setting to treat community-acquired pneumonia with risk factors for pseudomonas and hospital-acquired pneumonia as well as urinary tract infections. Known side effects include diarrhea, nausea, vomiting, pruritus, and headache. More severe reactions to cefepime are rare and include hypersensitivity reactions in patients with a penicillin allergy, neurotoxicity, encephalopathy, Clostridium-difficile-associated diarrhea, and the development of drug-resistant bacteria [1]. Cefepime-induced neurotoxicity has been associated with high morbidity and mortality and documented on objective testing with electroencephalogram (EEG) such as generalized periodic discharges, generalized rhythmic delta activity, and generalized spike-and-wave morphology [2]. The current report is regarding an 88-year-old female patient who was receiving cefepime to treat a urinary tract infection as an inpatient and subsequently developed a tremor in the extremities. Though neurotoxicity has been documented, serious adverse event tremor is not one of the known neurotoxic manifestations.

Cefepime's Food and Drug Administration (FDA) package insert warns of neurotoxicity manifesting as “encephalopathy (disturbance of consciousness, including confusion, hallucinations, stupor, and coma), aphasia, myoclonus, seizures, and non-convulsive status epilepticus” [3]. A review of published literature did not produce studies or case reports of tremors secondary to taking cefepime; therefore, this patient represents a rare example of cefepime-induced neurotoxicity and a relevant addition to the published literature on cefepime. A systematic review of ICU patients reported altered mental status, with reduced consciousness (47%), myoclonus (42%), and confusion (42%) to be the most common neurological manifestations of cefepime toxicity [4]. The review found one report where a patient experienced a disturbance involving extremity movement, which was, in that case, negative myoclonus [5].

## Case presentation

The patient is an 88-year-old female with a history of anemia, gastrointestinal bleeding, severe aortic stenosis, hypertension, hyperlipidemia, chronic heart failure, diabetes type 2, previous stroke with right-sided hemiparesis, previous deep vein thrombosis and pulmonary embolism, stage IV open sacral wound, and a right heel pressure sore who was brought to the hospital in January 2024 by her daughter due to an acute change in mental status.

In the Emergency Department, the patient underwent a CT brain without contrast, which was negative for any acute abnormality. Urine analysis was notable for white blood cells and leukocyte esterase, and the patient was admitted for antibiotic treatment of her urinary tract infection. Acute toxic metabolic encephalopathy presented as confusion to time and place and was likely secondary to the urinary tract infection. Admission labs were notable for leukocytosis, with a reported WBC count of 13,700 per microliter, consistent with an infectious process. Kidney function was normal with a creatinine of 1.02. Home medications were continued, including atorvastatin, irbesartan, furosemide, ezetimibe, aspirin, apixaban, amlodipine, and insulin.

The antibiotic regimen initially included intravenous vancomycin, metronidazole, and cefepime. Cefepime was not renally dosed at 2 grams, given intravenously twice daily. On admission day two, a physical exam revealed that the patient had developed a new low-frequency tremor in the left upper extremity and bilateral lower extremities that was found to be present at rest and improved with activity. Confusion present at admission had resolved. The right side was spared due to chronic hemiparesis. The patient reported no previous history of tremors. Urine cultures came back showing >100,000 Klebsiella, >100,000 Enterococcus faecalis, and >100,000 Enterococcus faecium, all of which were susceptible to cefepime at <1 mcg/ml. Metronidazole and vancomycin were discontinued at this time.

On the third day of admission, the patient was taking her home medications in addition to cefepime. Examination found continuous tremors of the left upper and lower extremities, including at rest. Out of concern that the tremor could be attributed to the cefepime, we discontinued it in favor of ciprofloxacin.

On day four of admission, the tremor was found to be improved and reduced in severity by approximately 50%. Urine culture sensitivities came back and the standing antibiotic regimen was replaced with linezolid and trimethoprim/sulfamethoxazole. Tremor severity further improved on day five of admission and was completely resolved by admission day six. Overall, it took three days off cefepime for the tremor to fully resolve.

## Discussion

This patient is the first reported to develop a tremor as a neurotoxic side effect from taking cefepime. Established side effects, which we previously discussed, are again shown in Table 1. Healthcare providers should be aware of tremors as a potential side effect and should consider discontinuing treatment with cefepime if their patient develops a new tremor within days of initiating treatment. Additional causes of the patient's tremor were considered and reasonably excluded, such as thyroid disorder, hypoglycemia, alcohol withdrawal, or as a consequence of other medications. Normal glucose, thyroid labs, and abstinence from alcohol made these reasons unlikely. Other hyperkinetic movements were considered such as myoclonus, but the rhythmicity of the patient's movements suggests classifying the movement as a tremor.

**Table 1:** Known adverse reactions in cefepime multiple-dose dosing regimens clinical trials in North America Source: [3] *Local reactions, irrespective of relationship to cefepime in those patients who received intravenous infusion (n=3048)

Adverse Reactions	
Incidence equal to or greater than 1%	Local adverse reactions (3%), including phlebitis (1.3%), pain and/or inflammation (0.6%)*; rash (1.1%)
Incidence less than 1% but greater than 0.1%	Colitis (including pseudomembranous colitis), diarrhea, erythema, fever, headache, nausea, oral moniliasis, pruritis, urticaria, vaginitis, vomiting, and anemia

Cefepime is renally excreted with a creatinine clearance of 74.0 (±15.0) mL/min, and research has shown a decrease in cefepime total body clearance as a function of creatinine clearance [3]. Dosage adjustment appropriate for patients' degree of renal impairment should be done for patients with renal dysfunction, those receiving hemodialysis, and geriatric patients with renal impairment having experienced serious adverse events after being given unadjusted doses of cefepime [6]. The safety of cefepime has been studied in the elderly population (65 and over) and there were no additional risks as long as the dosage was adjusted as appropriate if the patient’s creatinine clearance was 60 mL/min or less. The FDA recommends discontinuing cefepime and instituting appropriate supportive measures if neurotoxicity symptoms occur [3]. In the majority of cases, symptoms of neurotoxicity were reversible and resolved after discontinuation of cefepime and/or after hemodialysis [6]. In this patient with an admission creatinine of 1.02 and estimated GFR of 53, it is possible that the patient’s renal function contributed to more severe symptoms because cefepime was not renally dosed.

Elimination of cefepime has an average half-life of two hours and total body clearance of 120 (±8) mL/min in healthy volunteers and pharmacokinetics are linear at a therapeutic dose [3]. Previous research has found that toxic encephalopathy appears two to six days after cefepime administration and disappears three days after discontinuation of cefepime [7]. The mechanism of cefepime's neurotoxicity is related to its antagonism of gamma-aminobutyric acid (GABA). The patient reported here had a timeline consistent with the previous literature, with symptoms appearing after two days on cefepime and disappearing three days after stopping the medication. This suggests that the mechanism of our patient’s cefepime-induced tremor is the drug's neurotoxic properties, rather than a musculoskeletal or other metabolic etiology.

## Conclusions

This patient is the first reported to develop a tremor as a neurotoxic side effect from taking cefepime. The timeframe of symptom onset (two to six days) and resolution of symptoms after stopping treatment with cefepime (three days) is similar to previously published literature on the neurotoxic effects of cefepime. This suggests that the mechanism of the current patient’s cefepime-induced tremor is the drug's neurotoxic properties, rather than a musculoskeletal or other metabolic etiology. Healthcare providers should be aware of this potential side effect and consider discontinuing treatment with cefepime if their patient develops a new tremor within days of cefepime initiation.
